# International expert opinion on the considerations for combining vosoritide and limb surgery: a modified delphi study

**DOI:** 10.1186/s13023-024-03236-4

**Published:** 2024-09-17

**Authors:** Silvio Boero, Julia Vodopiutz, Mohamad Maghnie, Josep M. de Bergua, Ignacio Ginebreda, Hiroshi Kitoh, Micha Langendörfer, Antonio Leiva-Gea, Jason Malone, Philip McClure, Gabriel T. Mindler, Dmitry Popkov, Robert Rodl, Pablo Rosselli, Fabio Verdoni, Viktor Vilenskii, Aaron J. Huser

**Affiliations:** 1 Pediatric Orthopaedic and Traumatology Unit, IRCCS Istituto Giannina Gaslini, Genova, Italy; 2 Vienna Bone and Growth Center, Währinger Gürtel 18–20, Vienna, Vienna, 1090 Austria; 3 Department of Pediatrics and Adolescent Medicine, Division of Pediatric Pulmonology, Allergology and Endocrinology, Comprehensive Center for Pediatrics, Medical University of Vienna, Vienna, 1090 Austria; 4 Department of Pediatrics, IRCCS Istituto Giannina Gaslini, Genova, 16147 Italy; 5 Department of Neuroscience, Rehabilitation, Ophthalmology, Genetics, Maternal and Child Health, University of Genova, Genova, 16147 Italy; 6 Unidad Cirugía Artroscópica (UCA), Hospital Vithas Vitoria, Vitoria-Gasteiz, Spain; 7 Hospital Universitari Dexeus - Grupo Quirónsalud, Calle Sabino Arana, 5-19 - Planta 1, Barcelona, 08028 Spain; 8 Department of Orthopaedic Surgery, Aichi Children’s Health and Medical Center, 7-426, Morioka-cho, Obu, Aichi 474-8710 Japan; 9 Orthopedic Department of Kinderklinik Sankt Augustin, Arnold-Janssen-Straße 29, 53757 St. Augustin, Germany; 10 UGC Cirugía Ortopédica y Traumatología, Hospital Universitario Virgen de la Victoria, Instituto de Investigación Biomédica de Málaga (IBIMA)-Plataforma Bionand, Málaga, España; 11 Nemours Children’s Hospital – Florida, Orlando, FL USA; 12 Rubin Institute for Advanced Orthopedics, Sinai Hospital of Baltimore, 2401 W. Belvedere Avenue, Baltimore, MD 21215 USA; 13 Department of Pediatric Orthopaedics and Foot Surgery, Orthopaedic Hospital Speising, Speisinger Strasse 109, Vienna, 1130 Austria; 14 National Ilizarov Medical Research Centre for Traumatology and Ortopaedics, 6, M.Ulyanova street, Kurgan, 640014 Russia; 15 Universitätsklinikum Münster, Universitätsklinikum Münster, Albert-Schweitzer-Campus 1, Gebäude A1, Anfahrtsadresse: Albert-Schweitzer-Straße 33, 48149 Münster, Germany; 16 Fundación Cardio infantil Facultad de Medicina, Bogota, Colombia; 17 IRCCS ‘Galeazzi’ Orthopedic Institute, Vis Riccardo Galeazzi, 4, Milano, 20161 Italy; 18 St Petersburg State University Hospital, St Petersburg, Russia; 19 Paley Advanced Limb Lengthening Institute, West Palm Beach, Florida, USA

**Keywords:** Achondroplasia, Bone lengthening, Deformity correction, Modified Delphi process, Limb surgery, Management, Vosoritide

## Abstract

**Background:**

Achondroplasia is the most common form of skeletal disorder with disproportionate short stature. Vosoritide is the first disease-specific, precision pharmacotherapy to increase growth velocity in children with achondroplasia. Limb surgery is a standard approach to increase height and arm span, improve proportionality and functionality, as well as correcting deformities. The aim of this study was to gain expert opinion on the combined use of vosoritide and limb surgery in children and adolescents with achondroplasia.

**Methods:**

An international expert panel of 17 clinicians and orthopaedic surgeons was convened, and a modified Delphi process undertaken. The panel reviewed 120 statements for wording, removed any unnecessary statements, and added any that they felt were missing. There were 26 statements identified as facts that were not included in subsequent rounds of voting. A total of 97 statements were rated on a ten-point scale where 1 was ‘Completely disagree’ and 10 ‘Completely agree’. A score of ≥ 7 was identified as agreement, and ≤ 4 as disagreement. All experts who scored a statement ≤ 4 were invited to provide comments.

**Results:**

There was 100% agreement with several statements including, *“Achieve a target height, arm span or upper limb length to improve daily activities”* (mean level of agreement [LoA] 9.47, range 8–10), the “*Involvement of a multidisciplinary team in a specialist centre to follow up the patient”* (mean LoA 9.67, range 7–10), “*Planning a treatment strategy based on age and pubertal stage”* (mean LoA 9.60, range 8–10), and “*Identification of short- and long-term goals, based on individualised treatment planning”* (mean LoA 9.27, range 7–10), among others. The sequence of a combined approach and potential impact on the physes caused disagreement, largely due to a lack of available data.

**Conclusions:**

It is clear from the range of responses that this modified Delphi process is only the beginning of new considerations, now that a medical therapy for achondroplasia is available. Until data on a combined treatment approach are available, sharing expert opinion is a vital way of providing support and guidance to the clinical community.

**Supplementary Information:**

The online version contains supplementary material available at 10.1186/s13023-024-03236-4.

## Background

Achondroplasia is the most common form of skeletal disorder with disproportionate short stature, with an estimated prevalence of 3.72–4.6 per 100,000 births [1,2]. The condition is caused by a gain-of-function mutation in the gene for fibroblast growth factor receptor-3 (*FGFR3*), [3, 4] and 80% of cases occur *de novo.* [5, 6] At a physiological level the mutation leads to impaired endochondral ossification and bone growth, and results in characteristic disproportionate short stature [5], with projected adult height of 123–143 cm for males and 115–134 cm for females [7, 8]. Clinically, people with achondroplasia experience a variety of medical, functional, and psychosocial challenges across their lifespan – including complications such as foramen magnum stenosis, as well as spinal stenosis, kyphosis, pain, limb deformities, and ear, nose and throat (ENT) complications, among others. [5, 9–12].

Until recently, treatments for people with achondroplasia addressed complications and symptoms, but not the underlying condition. In some countries, elective surgical limb lengthening (LL) is considered a standard option in a selected group of patients to increase height and arm span, improve proportionality and functionality, and to correct deformities. In addition to lengthening of the leg bones to increase height, functionality and ability to perform activities of daily living, humeral lengthening improves functionality and self-care abilities [5, 13–17]. The average bone length increases achieved in people with achondroplasia who undergo LL range from 6 to 21 cm and may require multiple operations [18]. However, whilst there is a long history of surgical intervention in this patient group, there is currently no internationally accepted gold standard approach. A number of approaches, techniques and devices have been investigated for lower limb lengthening [15–22], from external fixators to internal lengthening nails [23–26]. Some advocate simultaneous extensive lengthening, whilst others opt for a series of more moderate procedures, which result in less trauma on soft tissues and joints [22], but require repeat surgeries. There are fewer publications on the strategies for upper limb lengthening alone, and studies proposing strategies for simultaneous lower and upper limb lengthening [15, 20, 21]. Timing of LL can vary, with procedures reported from early childhood to adolescence [5, 15, 21]. 

Opinion remains divided about whether LL is a suitable option for these patients [21], and many patient support groups are not in favour [27]. Opponents argue that LL does not address any of the more serious medical aspects associated with achondroplasia, and may put patients at risk of complications, including fracture, nerve damage, or unnecessary disability [18, 22, 28]. There is also conflicting evidence around the impact of LL on quality of life, with some studies reporting lower quality of life in those undergoing LL compared with non-lengthened patients [29]. Others cite that 85% are satisfied with their improved physical and psychological status as a result of LL surgery [30] and enjoy a greater quality of life [9, 31, 32]. Despite these controversies, the recent international consensus statement on the management of achondroplasia includes a recommendation on the possibility of children with achondroplasia undergoing LL, but stresses the need to balance all functional, physical, and psychosocial outcomes, and for surgery to be performed in a centre of excellence for achondroplasia [5]. Psychological assessment and appropriate support is advised before undertaking LL [5, 33]. 

A further consideration is whether LL has an impact on physeal growth, but this is also controversial. Some studies suggest this underlying biological process is not affected by LL [34], while others report that physeal growth may decrease and even stop after LL [35–39]. The amount of, and the age of the patient at, LL may impact this process. Song and colleagues report that partial physeal closure occurred in over 50% of patients with achondroplasia who underwent extensive limb lengthening [38]. This disturbance may depend on the specific procedure used. For example, data from a recent retrospective study show simultaneous bilateral femoral and tibial lengthening results in more physiological physeal disturbance effects (temporary or permanent cessation of growth as a result of compressive forces) than consecutive lengthening – although the difference was not statistically significant [40]. However, this technique may enable a more harmonious approach to LL, with less percentage of lengthening required per segment, as well as fewer complications.

Longitudinal bone growth is achieved by endochondral bone formation – a tightly controlled process stimulated by C-type natriuretic peptide (CNP), which activates the cAMP/protein kinase A pathway [41, 42]. This activation contributes to elongation of the hypertrophic zone in the growth plate [41]. Vosoritide is a modified CNP analogue that counteracts overactive FGFR3 signalling and stimulates endochondral bone growth [43]. It is the first disease-specific, precision pharmacotherapy to increase growth velocity in children with achondroplasia, and in clinical trials this novel agent showed an increase in annualised growth velocity (AGV) and improvements in body proportionality over 104 weeks [44–46]. Based on findings from the clinical trial programme, vosoritide is now approved for use in children with achondroplasia in several countries. The lower age limit varies by country with some able to prescribe in children aged ≥ 2 years and some ≥ 5 years, however, open physes are required to start and continue the medication [47, 48]. 

It has been argued that children with achondroplasia may not achieve full adult height with pharmacotherapies, since they are unlikely to regain the inhibited growth that preceded treatment initiation [21]. In light of this, there are many discussions and questions that need to be asked around the safety and potential efficacy of combining vosoritide and limb surgery. Key considerations include whether a patient can try both treatment approaches, and – if so – in what order, at what timepoints, and what factors will impact eligibility or suitability, such as the age of the patient at the start of vosoritide, and age at limb surgery. It is possible that surgical limb lengthening for children with achondroplasia may still be needed – but to a lesser extent, and only at skeletal maturity [21] or in those requiring deformity correction [49]. 

Since there are no data available to answer these questions, an international expert panel was convened to develop statements for consideration in the event that a patient and their clinician may choose to undertake a combination of vosoritide and limb surgery, or wish to make an informed choice between the two. This publication presents the collective opinion of the expert panel.

## Methods

### Study objective

A modified Delphi process [50, 51] was undertaken with the objective of gaining expert opinion on the combined use of vosoritide therapy and limb surgery in children and adolescents with achondroplasia. For the purposes of this study, ‘limb surgery’ includes surgical lengthening, corrective osteotomy, and guided growth.

In the absence of evidence-based data on the combined treatment in this group of patients, this Delphi process was undertaken to offer eminence-based guidance for clinical practice. Gaining consensus to provide recommendations was not an aim of the process. The statements identified are not intended as recommendations for management, they are considerations for treatment, based on the collective knowledge of international experts in the field. It is acknowledged that practices may vary between countries.

### Participants

The panel consisted of 17 clinicians from Austria (*n* = 2), Colombia (*n* = 1), Germany (*n* = 2), Italy (*n* = 3), Japan (*n* = 1), Russia (*n* = 2), Spain (*n* = 3), and the United States of America (*n* = 3). There were 15 orthopaedic surgeons (SB, JBD, IG, AH, HK, ML, ALG, JM, PM, GM, DP, RR, PR, FV, VV), one paediatric endocrinologist (MM) and one paediatric and clinical geneticist (JV). All are experts in the care of patients with achondroplasia.

### Study design

A modified Delphi process was used to develop and establish levels of agreement on statements pertaining to the combined use of vosoritide and limb surgery [50, 51]. An independent methodologist (DHJ) was consulted prior to the initiation of the study to provide advice on study design, extraction of statements from advisory board data and generation of Delphi survey. DHJ also provided oversight during the collection and write-up of the data.

Two meetings were held: a face-to-face meeting in February 2020, with the aim of exploring global perspectives on limb lengthening in achondroplasia, and a virtual meeting in April 2022, with the aim of improving understanding of the considerations of vosoritide treatment in patients undergoing LL surgery. Both meetings were organised and funded by BioMarin Pharmaceutical Inc. BioMarin had no input into the Delphi process or manuscript development.

The research question for the modified Delphi process was developed by the research team and verified by the expert panel *a priori*.

### Analysis and delphi statement generation

**Fig. 1:**
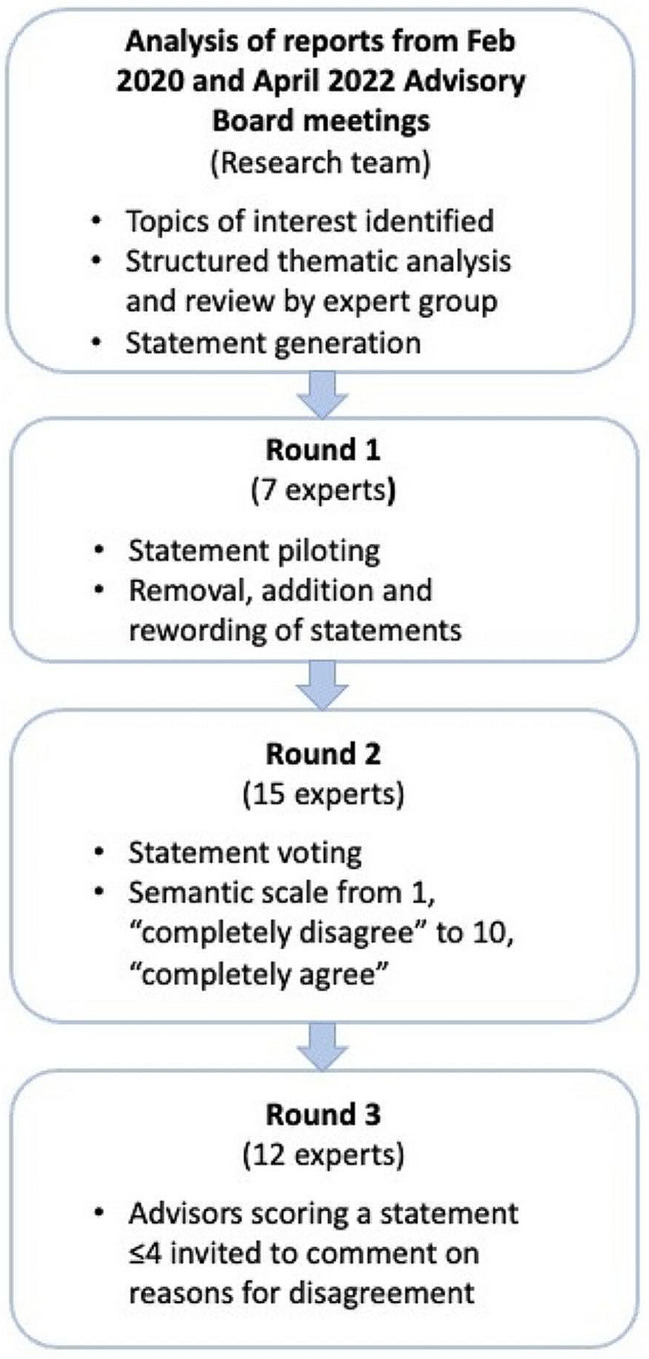
Study design

Data gathered during both meetings were audio recorded, transcribed verbatim and analysed. Four categories of interest were identified. Section 1: The knowns and unknowns of vosoritide, limb surgery, and combination of the two, that should be taken into consideration in all treatment planning and decision-making; Sect. 2: Considerations for patients who may accept either vosoritide or limb surgery, separately, or in any combination; Sect. 3: Considerations for patients who have already started limb surgery where vosoritide is being considered; and Sect. 4: Considerations for patients already prescribed vosoritide where limb surgery is being considered. A structured thematic analysis of the meeting reports was carried out under these four categories (DHJ) and used as the basis for the Delphi process and generation of statements, outlined in Fig. 1.

Four themes were identified for the factual statements in Sect. 1 (treatment planning, impact of vosoritide, guided growth, and concerns) and five common themes identified for Sects. 2–4 (drivers and goals, pre-treatment/surgery considerations, timing/age considerations, contraindications, and follow up).

A total of 120 statements were generated from the thematic analysis and Round 1 statement piloting was carried out. A total of seven members of the expert panel reviewed the statements, made changes to wording, removed any they considered unnecessary, and added any that they felt were missing. No votes were cast at this stage. At Round 2, 15 of the expert panel voted on the 97 statements in Sects. 2–4. Section 1 was excluded from voting as these statements are accepted as facts (according to knowledge available in January 2023). The group rated each statement on a ten-point rating scale where 1 was ‘Completely disagree’ and 10 ‘Completely agree’. A score of ≥ 7 was identified *a priori* as agreement with the statement, and a score ≤ 4 was identified *a priori* as disagreement with the statement [52]. In Round 3, all experts (*n* = 12) who scored a statement ≤ 4 were invited to provide comment on their reasons for disagreeing. As the aim of the study was not to find consensus, no further vote was carried out. All data were compiled by a member of the research team and underwent independent quality control to ensure accurate compilation of the votes. Mean level of agreement (LoA), range and percentage of experts in agreement with each statement were calculated. Further statistical tests were deemed inappropriate given the small number of experts voting.

## Results

The factual statements identified are shown in Table 1. These statements cover the knowns and unknowns of vosoritide and limb surgery, as of January 2023. These statements should be taken into consideration in all treatment planning and decision-making regarding vosoritide and/or limb surgery.

Statements pertaining to patients who are considering either vosoritide or limb surgery are outlined in Table 2. The expert panel agreed through discussion while developing the manuscript that a multidisciplinary approach to management will be needed to effectively monitor patients receiving dual therapy. Assessments to monitor limb surgery and/or vosoritide therapy should be carried out by the relevant member of the multidisciplinary team, for example the orthopaedic surgeon to monitor bony parameters, with the vosoritide-prescribing physician carrying out assessments such as blood work, body proportions, and anthropometry. Clear communication amongst the team will be needed to ensure all assessments are completed. An example of a suggested schedule of assessments for monitoring patients receiving vosoritide and limb surgery in any combination is shown in Table 3.

Statements in Sects. 3 and 4 should be considered in addition to those relating to any combined treatment approach shown in Table 2. The statements relating to patients who have already started limb surgery where vosoritide is being considered are shown in Table 4, and for patients already prescribed vosoritide where limb surgery is being considered in Table 5.

**Table 1:** Factual statements (as of January 2023) that should be taken into consideration in all treatment planning and decision-making regarding vosoritide and limb surgery

Treatment planning
a. There are no data available on the use of vosoritide during the surgical limb lengthening process b. There are no data to support a combined treatment approach, in humans or from animal models c. There are no data to contradict a combined treatment approach, in humans or from animal models d. There are no data or clear guidance on how to use both approaches to get the optimal result e. There are no identified additional contraindications to those already known about vosoritide therapy or surgical limb lengthening alone f. A lack of knowledge of the additional risk of combined treatments may impact clinical decision-making
Impact of vosoritide
g. The effect of vosoritide on final adult height and the end of growth is unknown h. The long-term safety of vosoritide is unknown i. Whether early use of vosoritide therapy will result in less disproportion, or need for deformity correction is unknown j. Whether vosoritide has an impact on the consolidation of subsequent surgical limb lengthening is unknown k. Whether there are any harmful effects of the combination of treatments on the bone length and segment is unknown l. The contribution of endochondral or periosteal bone during treatment with vosoritide is still to be understood m. Whether vosoritide has an effect on the physes when combined with limb surgery is unknown n. Whether vosoritide is involved in healing, callus formation, or delayed healing is unknown o. There is a risk of inhibition of growth plate function with extensive lengthening p. The effect of vosoritide on deformities of the spine and limbs is unknown q. Devices used for lengthening should avoid any mechanical damage/injury of physes r. Whether there are differences between upper and lower limb surgery with concomitant use of vosoritide is unknown
Guided growth
s. Similar to surgical lengthening or osteotomy, it is feasible to administer vosoritide during guided growth surgery t. If growth is improved by vosoritide, guided growth procedures may be faster and require earlier removal of implants; improvement of growth may allow correction of more significant deformity with growth modulation u. The effect of vosoritide when it comes to the efficacy of guided growth constructs is unknown v. The effect of vosoritide on deformity recurrence following guided growth procedures is unknown
Concerns
w. Whether surgical lengthening would weaken or strengthen the potential height gain of vosoritide x. Whether there are specialist centres that offer both treatments y. Whether puberty arrest would be an option to gain more time for vosoritide treatment z. How to counsel patients/families in the best way aa. The potential (currently unknown) side effects of stopping vosoritide treatment during episodes of surgical lengthening

**Table 2:** For patients who are considering either vosoritide or limb surgery (where ‘limb surgery’ includes surgical lengthening, corrective osteotomy, or guided growth)

Statement	Level of agreement, mean (SD)	Range	% agreement*
Theme 1. Drivers and Goals. *When considering combining vosoritide and limb surgery the goals are to …*			
Achieve a target height, arm span or upper limb length to improve daily activities	9.47 (0.64)	8–10	100%
Attain as much benefit as possible from all available therapies	9.07 (1.71)	4–10	93%
Minimise the total number of surgical interventions to increase height	8.93 (1.62)	5–10	87%
Achieve the maximum possible treatment duration of vosoritide, to maximise height and potential benefits beyond height†	8.87 (1.77)	5–10	87%
Achieve maximum height with a combination of vosoritide and surgical lengthening	8.87 (1.25)	7–10	100%
Improve functionality to achieve maximum age-related independence in personal hygiene and activities of daily living	8.73 (1.58)	5–10	93%
Achieve height gains in older children in whom vosoritide alone will not meet the target	8.53 (1.68)	5–10	87%
Improve functionality to enable participation in all age-related activities (e.g., sport, social activities)	8.40 (1.59)	6–10	80%
Explore the opportunity for deformities to be corrected faster by combining vosoritide with guided growth surgery	8.40 (2.23)	5–10	80%
Improve physical, emotional, and social domains	8.27 (2.09)	3–10	80%
Focus on functional improvement, not height	8.07 (1.98)	5–10	67%
Improve proportionality	8.00 (1.77)	5–10	73%
Explore the potential for reciprocal amplification of outcomes in the combination of vosoritide with surgical limb lengthening	8.00 (1.85)	4–10	80%
Achieve maximum growth possible with vosoritide first, thereby minimising the need for, or total number of surgical interventions needed to increase height	7.40 (3.40)	1–10	67%
Improve the self-esteem of the patient	7.40 (2.87)	1–10	67%
Explore opportunities for further benefits of vosoritide, beyond height gain	7.33 (3.06)	1–10	73%
Obtain a sufficient increase in the length of the tibia and femur with vosoritide to enable surgical lengthening with implantable, lengthening nails after fusion of the physis	7.27 (2.91)	1–10	67%
Theme 2. Pre-treatment considerations. *Considerations prior to initiation of combined vosoritide and limb surgery should include …*			
Treatment planning			
Involvement of a multidisciplinary team in a specialist centre to follow up the patient	9.67 (0.90)	7–10	100%
Previous surgical limb lengthening is not a contraindication to receiving vosoritide	9.67 (0.82)	7–10	100%
Planning a treatment strategy based on age and pubertal stage	9.60 (0.83)	8–10	100%
Identification of short- and long-term goals, based on individualised treatment planning	9.27 (1.16)	7–10	100%
Management of patient/family expectations	9.07 (1.62)	5–10	87%
Adherence to pre-treatment protocols, as for any therapeutic or surgical approach	9.00 (1.85)	3–10	93%
Establishing the age-dependent therapeutic window for the use of vosoritide	8.73 (2.28)	3–10	87%
Imaging to exclude foramen magnum stenosis prior to surgical intervention	8.60 (2.59)	1–10	80%
Identification of the best subgroup of candidates for treatment	8.40 (2.16)	3–10	80%
Potential impact of vosoritide on limb surgery practices			
Reviewing the need for surgical limb lengthening procedures in patients who achieve target height and proportionality	9.60 (0.91)	7–10	100%
Reducing the need for extensive, or multiple rounds of, surgical limb lengthening	9.53 (1.36)	5–10	93%
Assessing the feasibility of dual therapy given the different modes of action of vosoritide and surgical limb lengthening	9.07 (1.44)	6–10	87%
Obtaining an understanding of the different modes of action of surgical limb lengthening and vosoritide	8.87 (1.46)	5–10	93%
The reassessment of surgical limb lengthening strategies given the inclusion of vosoritide	8.73 (2.40)	1–10	93%
Data indicate that mice with achondroplasia showed significantly better new bone formation than wild type mice; there are some concerns over the suppression of FGFR3 signalling by vosoritide during limb lengthening	7.20 (2.78)	1–10	67%
That combined vosoritide and limb surgery could add stress to the physis	5.80 (2.86)	1–10	40%
Decision-making process			
Discussion of the potential risks and benefits of all options with the patient and family as part of the decision-making process	9.87 (0.35)	9–10	100%
Supporting the decision-making process by providing factual and evidence-based information	9.47 (1.19)	6–10	93%
Making the decision to initiate vosoritide or surgical limb lengthening on an individual basis	9.33 (1.18)	7–10	100%
Understanding the safety of any combination of vosoritide and surgical limb lengthening prior to initiation of a dual approach	9.07 (2.02)	3–10	87%
Consideration of the emotional, time and economic burden for families of both approaches	9.00 (2.33)	1–10	93%
Involving at least the prescribing clinician, the orthopaedic surgeon, and a psychologist in the multidisciplinary team	8.47 (2.64)	1–10	80%
An informed, shared decision-making process, involving the patient (if age-appropriate)/family and a multidisciplinary team including a psychologist or psychiatrist	8.33 (2.64)	1–10	73%
Ensuring psychological support is available to the patient and family prior to and during either vosoritide therapy or surgical limb lengthening	7.93 (2.99)	1–10	80%
Theme 3. Timing *Considering the timing of a dual treatment approach, vosoritide should be …*			
Prescribed first, and surgical limb lengthening discussed with patients and families in an age-appropriate way, as needed to achieve treatment goal	8.33 (2.53)	1–10	80%
Prescribed as early as possible to achieve maximum effect and to reduce future surgical treatment and associated complications	8.33 (2.47)	1–10	80%
Considered in the case of patients waiting to receive surgical limb lengthening	8.27 (1.87)	3–10	93%
Prescribed prior to limb surgery, with the option to add surgical interventions once outcomes achieved with vosoritide can be assessed	7.27 (3.15)	1–10	73%
Initiated at any stage of the surgical limb lengthening process	6.27 (2.84)	1–10	40%
Theme 4. Contraindications *Combined vosoritide and limb surgery should NOT be considered if …*			
The patient and their family are not motivated to accept the treatments and necessary follow ups	9.87 (0.52)	8–10	100%
Any contraindications exist either for surgical interventions or medical therapy	9.47 (1.13)	6–10	93%
Family support is not in place	8.93 (2.28)	3–10	87%
There is a history of poor compliance	8.80 (2.40)	1–10	93%
It is difficult to assess growth potential remaining	7.20 (2.81)	1–10	60%
Ongoing surgical limb lengthening is in the distraction phase	5.40 (3.48)	1–10	53%
Theme 4. Follow up *Considerations for follow up should include …*			
A structured regime specific to the monitoring of both therapies	9.53 (1.13)	6–10	93%
Appropriate frequency of follow up according to local protocols	9.33 (1.29)	6–10	93%
Adherence to local protocols for surgical lengthening and vosoritide therapy	9.27 (1.44)	6–10	87%

*% of advisors scoring ≥ 7; †within the indication of vosoritide (while physes are open)

**Table 3:** A suggested example of assessments to monitor patients receiving vosoritide and limb surgery in any combination

Assessment	Before treatment		During treatment		After treatment	
	Essential	Desirable	Essential	Desirable	Essential	Desirable
Physical examination						
Anthropometry (which may include height; growth curve monitoring; sitting height and arm span; weight; body mass index; waist, hip, chest, and head circumference)	x		x		x	
Body proportions	x		x		x	
Bone assessment (which may include length, AGV)	x		x		x	
Functional recovery (including assessment of ADL, personal hygiene, age-related participation in sports and social activities)	N/A			x		x
HRQol variables (incl. PROs)	x		x		x	
Radiological assessments						
Radiological variables (which may include lower limb alignment, spine parameters)	x		x		x	
Annual X-Ray to assess bone formation		x	x		x	
Bone age (Dimeglio method, X-Ray of olecranon)	x		x		x	
Quality of callus*	N/A		x		x	
Surgery-specific assessments						
Deformity assessment†	x		x		x	
Soft tissue evaluation‡	x		x		x	
Joint stability‡	x		x		x	
Muscle function¶		x		x		x
Range of motion (may include gait study)	x		x		x	
Bone healing*	N/A		x		x	
Safety						
Safety (including assessment of medical and surgical complications**)	x		x		x	
Complementary assessments						
Physiotherapy	x		x		x	
Blood and urine parameters		x		x		x
Whole spine MRI		x				x

*Assessed by a validated method, such that by as Venkatesh et al.; [60] †May include clinical, radiographic, or gait examination; ‡Based on clinical examination; ¶ Muscle strength is graded according to the Medical Research Council (MRC) scale with zero being no contraction and five representing normal strength^61^; **Assessed by Paley [62], or alternative appropriate validated assessment [63]

**Table 4:** For patients who have already started limb surgery where vosoritide is being considered, in addition to considerations in Sect. 2

Statement	Level of agreement, mean (SD)	Range	% agreement*
Theme 1. Drivers for adding vosoritide *For patients who have already started limb surgery, the addition of vosoritide should be considered when …*			
The patient has already started surgical limb lengthening prior to the approval of vosoritide in their country, and wishes to try a non-surgical approach	8.47 (1.64)	5–10	87%
The expected final height with limb lengthening alone is not sufficient, and there is a desire to avoid further lengthening and related complications	8.20 (1.90)	5–10	80%
The expected final height with limb lengthening alone is not sufficient	7.60 (2.82)	1–10	67%
The patient/family want a greater chance of increased height	7.53 (2.29)	4–10	60%
Theme 2: Pre-treatment considerations *For patients who have already started limb surgery, considerations prior to initiation of vosoritide should include …*			
Managing the expectations of the patient/family on the potential effects of vosoritide on spontaneous growth of the forearm, humerus, or lower limbs	8.80 (1.78)	5–10	80%
Identification of the most appropriate dosage based on the weight of the patient	8.60 (2.97)	1–10	87%
Whether vosoritide may act as a substitute for further surgical limb lengthening	8.00 (2.73)	1–10	80%
Theme 3: Timing/Age considerations *For patients who have already started limb surgery, considerations for the timing of initiation of vosoritide should include …*			
Whether there is sufficient residual growth for the patient’s age	9.73 (0.59)	8–10	100%
The effect it may have on the final height for patients in adolescence	9.60 (0.91)	7–10	100%
That vosoritide can be initiated in patients already undergoing limb surgery	7.53 (2.53)	3–10	67%
That both vosoritide and surgical interventions could be prescribed concomitantly	7.07 (2.87)	1–10	60%
That vosoritide can be added at any stage of the limb lengthening process	6.93 (3.03)	1–10	60%
Whether the range of motion has been recovered	5.67 (3.87)	1–10	53%
Whether the frame has been removed	5.00 (3.48)	1–10	40%
Theme 4: Contraindications *For patients who have already started limb surgery, vosoritide should NOT be considered if …*			
There is a history of poor compliance	8.27 (2.81)	1–10	80%
Family support is not in place	8.00 (3.12)	1–10	73%
Theme 5: Follow up *Considerations for follow up should include …*			
Adherence to local protocols for monitoring of both treatment options	9.53 (1.13)	6–10	93%
Checking patient compliance with daily vosoritide injections	8.67 (1.80)	5–10	87%
Increased frequency of monitoring after initiation of vosoritide than usual surgical limb lengthening protocols	7.40 (2.59)	1–10	53%

*% of advisors scoring ≥ 7

**Table 5:** For patients already prescribed vosoritide where limb surgery is being considered, in addition to considerations in Sect. 2

Statement	Level of agreement, mean (SD)	Range	% agreement*
Theme 1. Drivers for adding limb surgery *For patients already prescribed vosoritide, the goals of limb surgery are to …*			
Improve functional limitations	9.67 (0.72)	8–10	100%
Correct deformity	9.40 (0.99)	7–10	100%
Increase height, lower and upper limb length beyond that achieved with vosoritide alone	9.20 (1.08)	7–10	100%
Theme 2: Pre-surgery considerations *For patients already prescribed vosoritide, considerations prior to initiation of limb surgery should include …*			
Assessing the commitment of the patient/family to undergo limb surgery	9.67 (0.72)	8–10	100%
Assessing the motivation behind the patient/family’s perceived need for limb surgery	9.53 (0.74)	8–10	100%
Establishing the growth expectations of the individual patient	9.47 (0.83)	8–10	100%
Establishing the amount of lengthening needed to achieve treatment goals in a safe manner	9.33 (1.18)	6–10	93%
Assessment of the age at which limb surgery is initiated	9.33 (1.29)	6–10	93%
Adherence to a protocol of pre-surgery assessments	9.20 (1.32)	6–10	93%
Assessing patient age, pubertal stage, open physes and the length of the bone segments	9.13 (1.36)	6–10	93%
Providing estimates of achievable outcomes to patients, based on available data and knowledge. This information will inform decisions to undergo additional lengthening, or to wait until the effect of vosoritide therapy is known	8.87 (1.85)	3–10	93%
Selecting the most appropriate type of surgery based on the drivers for adding limb surgery (for example, deformity correction vs. guided growth vs. surgical limb lengthening)	8.80 (2.37)	1–10	93%
Imaging of the spinal canal and foramen magnum to exclude anaesthetic-relevant risk factors	8.53 (2.47)	1–10	87%
Ensuring the maximum target length can be gained	7.73 (1.75)	4–10	80%
Establishing the risk factors for inhibition of growth zone function	7.73 (2.58)	2–10	67%
Theme 3: Timing/Age considerations *For patients already prescribed vosoritide, considerations for the timing of initiation of limb surgery should include …*			
Individualised decision-making whether to cease vosoritide during surgery	8.33 (2.58)	2–10	80%
Establishing that the maximum growth has been achieved with vosoritide treatment (the least traumatic surgical options can then be considered)	7.27 (2.96)	1–10	67%
Whether there is sufficient residual growth	5.87 (3.09)	1–10	33%
Theme 4: Contraindications *For patients already prescribed vosoritide, limb surgery should NOT be considered if …*			
The patient demonstrates a lack of motivation for limb surgery	9.27 (2.31)	1–10	93%
The patient is not a suitable candidate for surgical limb lengthening	9.00 (2.56)	1–10	87%
Theme 5: Follow up *Considerations for follow up should include …*			
Collecting data on new bone generation^†^	9.73 (0.59)	8–10	100%
Adherence to local protocols for monitoring of both treatment options	9.47 (1.13)	6–10	93%

*% of advisors scoring ≥ 7; † To assess whether vosoritide has an impact on the quality of the new bone growth

## Discussion

### General considerations

The expert panel provided a range of opinions on the statements generated from the thematic analysis. The breadth of response demonstrates that while there are some accepted facts relating to a combined vosoritide and limb surgery approach (Table 1), in the absence of published evidence most considerations are based on experience and beliefs. Some differences in opinion may be the result of different healthcare systems, levels of experience with vosoritide or limb surgery, cultural differences, or the specialty of the expert. Achondroplasia is a complex condition, requiring lifelong management by a range of specialities [5, 33]. Effective communication among the multidisciplinary team, and sharing knowledge and early experiences of combining medical and surgical options will support the clinical community as they are faced with decisions in clinical practice.

### Considering the knowns and unknowns of combining vosoritide and limb surgery

To date there are no published data available on the use of vosoritide in combination with limb surgery. The expert panel wanted to separate facts from opinion, so collated the knowns and unknowns in Table 1. There are no data to either support or contradict a combined approach, neither is there published guidance on how to achieve optimal outcomes. Until evidence is available, there is no definitive answer to the questions that may arise when combining vosoritide therapy and limb surgery.

While there are data on vosoritide available from the clinical trial programme [44–46], knowledge gaps remain regarding long-term safety and efficacy, impact on proportionality and deformity correction, and on the impact of vosoritide on bone healing, callus formation and the physes. It is also unknown whether vosoritide and limb surgery have a positive or detrimental effect on one another in relation to potential height gain. One question that may arise in clinical practice is whether there are specialist centres that offer both treatments. The expert panel felt strongly, in line with recent guidance [5, 33], that both treatments should be offered in specialist centres with multidisciplinary experience of achondroplasia, and in addition, that having both treatments available in one place is essential to enable effective analysis of a combined approach. It is noted, however, that this may not be feasible, and is dependent on the individual country or centre setting.

For patients receiving vosoritide who are eligible for limb surgery, there are no data on the effect of pausing treatment whilst undergoing surgery. It is feasible that there is potential for antibody production if vosoritide is administered in a stop-start manner, but there are no data to support or contradict this hypothesis. A reduced impact on the final height may also be observed. Pausing vosoritide may create psychosocial stress for young children when restarting injections, as evidence from other disease areas indicate that previous experiences can affect willingness to accept an intervention [53]. What can be surmised is that as an elective therapy to augment growth, there will be no effects that are detrimental to a patient’s health should vosoritide be paused or stopped during limb surgery.

### Considering vosoritide, limb surgery, or both, in any combination

The majority of statements developed from the thematic analysis were relevant to vosoritide and limb surgery in any combination, regardless of which was initiated first (Table 2). The expert panel were keen to capture these as overarching statements to be taken into consideration in any instance in which vosoritide and limb surgery may be combined.

### Drivers and goals

It was emphasised by several experts that all treatment options must be focused on achieving the best proportion and functionality for patients and never the greatest height gain. Limb surgery in achondroplasia may be undertaken for many reasons, not only to achieve an increase in height. Improving proportionality and correcting deformities are key goals of limb surgery in this group of patients, and techniques including corrective osteotomy and guided growth are frequently used. Experts speculated, based on experience in other disease areas [54], that deformity correction rate using guided growth in combination with medical therapy may be improved. Collecting data on a subgroup of patients undergoing guided growth in combination with vosoritide would be beneficial to understanding whether there is a correlation between correction rate and medical therapy.

Experts strongly agreed that a goal of combined vosoritide and limb surgery is to *“Achieve a target height, arm span or upper limb length to improve daily activities”* (LoA 9.47, range 8–10). *“Attain as much benefit as possible from all available therapies”* also saw strong agreement with 93% of experts scoring ≥ 7. Anecdotally there are expectations in the achondroplasia community that vosoritide may have an impact on medical complications, in addition to augmenting linear growth. The expert panel largely agreed that a key goal of combined therapy is to “*Achieve the maximum possible treatment duration of vosoritide, to maximise height and potential benefits beyond height*” (LoA 8.87, range 5–10). While there are no data to prove or disprove the potential benefits beyond height, maximising treatment duration (providing no adverse events are identified) enables patients to experience as many potential benefits as possible. However, there were a range of opinions on the statement “*Explore opportunities for further benefits of vosoritide, beyond height gain*” (LoA 7.33, range 1–10), with some experts commenting that it is not appropriate to explore potential outcomes in clinical practice.

The goal to *“Achieve maximum growth possible with vosoritide first, thereby minimising the need for, or total number of surgical interventions needed to increase height”* (LoA 7.40, range 1–10) was not universally agreed, with two of the panel scoring this statement a 1 (completely disagree). The experts argued that deformities such as maltorsion and varus need to be corrected during growth, and while cases of simple lengthening could wait until the end of growth, other limb surgeries are likely to be required prior. For some patients, and in some cultures, height is important from a young age for social reasons, so waiting for the effect of vosoritide before considering surgical lengthening may not be desirable. However, one expert felt that the staged approach of seeking maximum benefit of vosoritide before adding surgical treatments may provide fewer safety concerns.

The potential impact of vosoritide on the choice of surgical approach was not considered a key goal of combined treatment, with the statement *“Obtain a sufficient increase in the length of the tibia and femur with vosoritide to enable surgical lengthening with implantable, lengthening nails after fusion of the physis”* receiving a range of scores (LoA 7.27, range 1–10).

It is important to be cognisant of the fact that in addressing physical aspects of achondroplasia, other domains may also be affected. For example, improving proportionality can directly impact on emotional and psychosocial domains, as a patient becomes more independent, more able to carry out self-care tasks, and may subsequently increase in confidence [9], however there is no evidence for a combined approach improving emotional and social domains. Due to this lack of evidence, improving the self-esteem of the patient, while identified as important, was not a widely agreed goal of combined vosoritide and limb surgery (LoA 7.40, range 1–10).

### Pre-surgery considerations

Selecting the most suitable candidates for vosoritide or limb surgery was considered by some on the panel to be the most important part of treatment planning, although one expert considered that, as achondroplasia is a relatively homogeneous disease, there are not subgroups of patients for whom treatment is most suited, and that vosoritide should be available to all patients within the product licence. In line with recent recommendations [5, 33], “*Involvement of a multidisciplinary team in a specialist centre to follow up the patient* “(LoA 9.67, range 7–10) received 100% agreement.

Excluding the presence of foramen magnum stenosis (FMS) is important prior to limb surgery, and consensus recommendations state that foramen magnum stenosis, more common in the first 2 years of life, benefits from early intervention [5]. Many experts considered the presence of clinically relevant FMS to be an exclusion criterion for limb surgery due to the risk from intubation and long periods of anaesthesia needed in some limb surgeries [55]. 

Despite a high level of agreement among most authors, the statement *“The reassessment of surgical limb lengthening strategies given the inclusion of vosoritide”* (LoA 8.73, range 1–10) received strong disagreement from one, based on the lack of evidence on which to base such a reassessment. There was some disagreement about the potential impact of vosoritide on bone formation, with a range of 1–10 for the statement *“Data indicate that mice with achondroplasia showed significantly better new bone formation than wild type mice; there are some concerns over the suppression of FGFR3 signalling by vosoritide during limb lengthening”* (LoA 7.20, range 1–10). Similarly, there was a lack of agreement on the statement *“That combined vosoritide and limb surgery could add stress to the physis”* (LoA 5.80, range 1–10). It is important to acknowledge that ‘limb surgery’ as discussed herein encompasses many different techniques and considerations which may or may not add stress to the physis. For example, for patients aged < 10 years the stress to the physis could be more than in older children, per published literature [39]. In addition, the amount of lengthening and the lengthening strategy may impact the level of stress applied to the physis. As such, this statement was considered by some to be too broad to be able to agree upon.

The inclusion of psychological support prior to and during vosoritide or surgical limb lengthening was largely agreed upon (LoA 7.93, range 1–10), although it was noted that psychological support may not be available in all countries or institutions, so may not be a key factor for all patients and clinicians in the decision-making process.

### Timing

There was some disagreement among the panel as to the point at which vosoritide could be added to an ongoing planned limb surgery, with some suggesting waiting for consolidation of the bone before starting with vosoritide, and some completely disagreeing with a sequential approach. Some experts argued that patients undergoing surgical limb lengthening may experience extensive pain, physical and psychological stress in the first 2–3 months, as well as receiving concomitant treatment with analgesics and physiotherapy, so vosoritide may not be a priority at this time.

### Contraindications

A possible contraindication to a combined treatment approach could be “*Ongoing surgical limb lengthening is in the distraction phase*” (LoA 5.40. range 1–10). This statement was added at Round 1 (statement piloting). One expert outlined that there are increased forces related to traction of the muscles and other soft tissues, especially during the distraction phase, which acts as compression on the growth plates. In a combined vosoritide and limb surgery approach, the stimulated function of physes under compression forces could, theoretically, be compromised. There are data to indicate a decrease in the distraction forces by the end of the fixation phase [56–59]. As such, it may be feasible that if surgical limb lengthening is undertaken first, vosoritide could be added at the end of distraction, during the early healing phase.

Family support during a combined treatment approach was seen as important, with lack of support agreed upon as a contraindication (LoA 8.93, range 3–10). It is usual to involve the family in the preparation for limb surgery or medical therapy, and in some cases, families will temporarily relocate to be close to the treating centre prior to surgery and to facilitate the immediate post-surgery follow up. Similarly for patients and families receiving medical therapy, attendance at a centre to understand the therapy and learn how to administer it safely is not unusual. It should be noted that while this is optimal, it may not be feasible in all healthcare settings.

### Considering the addition of vosoritide for patients who have already started limb surgery

There was general agreement with the pre-treatment consideration *“Whether vosoritide may act as a substitute for further surgical limb lengthening”* (LoA 8.00, range 1–10), however this will be dependent on the goals of the patient; if the goals for lengthening are modest, vosoritide may be a suitable substitute. Vosoritide may not be a substitute for surgical lengthening in patients with a greater lengthening goal, however, or in adolescents whose residual growth may not enable enough time to see a large effect with vosoritide.

There were differences in opinion on “*Whether range of motion has been recovered*” (LoA 5.67, range 1–10) is a pre-treatment consideration for adding vosoritide in patients who have already started limb surgery. Experts argued that range of motion is independent of the growth-promoting effects of vosoritide and that lack of recovery of range of motion should not preclude initiation of vosoritide. Similarly, for the statement “*Whether the frame has been removed*” (LoA 5.00, range 1–10); this was considered to be independent of vosoritide and should not therefore impact on its initiation. It should be noted that other devices may be used for surgical lengthening, and that this statement infers only to cases of lengthening with external fixators. The protocol for initiating vosoritide in patients who have already started limb surgery may vary between individual centres; until data and recommendations are available, differences in strategy are to be expected.

### Considering the addition of limb surgery for patients who have already started vosoritide

For patients receiving vosoritide, the pre-limb surgery consideration *“Ensuring the maximum target length can be gained”* (LoA 7.73, range 4–10), was found to be confusing as lengthening can be initiated before the extent of gains from vosoritide are evident. *“Establishing the risk factors for inhibition of growth zone function”* was considered to be a challenging statement by some as it is hard to identify how these risk factors could be established. A lengthening procedure added for patients who have already started vosoritide should avoid any injury (damage) of open growth zone (e.g., lengthening over transphyseal device–transphyseal elastic nailing or telescopic rod).

With regards to the timing of limb surgery in patients receiving vosoritide, a number of experts did not agree that consideration should be given to *“Whether there is sufficient residual growth”* (LoA 5.87, range 1–10). Whether the growth plates are open is not an important factor when considering limb surgery – lengthening procedures can be undertaken in skeletally mature patients. However, it is important to better understand the potential final height achievable with vosoritide to assess whether the patient really needs a surgical intervention. Insufficient residual growth alone was not considered to be a barrier when discussing limb surgery with a patient receiving vosoritide. There is also an argument that it is not necessary to wait until completion of growth either with or without vosoritide before considering limb surgery if the physician and patient agree it is a good option for them. However, it was deemed important to inform the patient of a lack of quality data when deciding *“…to undergo additional lengthening or wait until the effect of vosoritide is known”* (LoA 8.87, range 3–10).

### Limitations

The expert panel were representative of eight countries, from four continents, with different healthcare systems and processes for accessing vosoritide. These differences were not explored during statement generation in this modified Delphi process, although one author speculated that reimbursement of a combined approach may not be problematic as deformity correction can be carried out at the same time as surgical height augmentation. It may be beneficial in future work to assess any differences in the key considerations for combined treatment depending on healthcare system and access to treatment.

The panel consisted of surgeons and clinicians from expert centres, with a vast experience in limb surgery, and with early access to vosoritide. Their views may not reflect clinical practice in all centres managing people with achondroplasia.

There were aspects of combined limb surgery and vosoritide that were not addressed in this process, as the implications are many. Topics not included but that warrant further discussion and investigation include what impact vosoritide, if prescribed first, could have on different techniques used for surgical lengthening and whether it may allow less traumatic techniques to be employed, the amount of lengthening per segment, and specific aspects of surgery.

## Conclusion

This modified Delphi process outlines key considerations for the use of vosoritide in combination with limb surgery in clinical practice. It is clear from the range of responses and the aspects of surgical intervention not covered in this study, that this modified Delphi process is only the beginning of new considerations now that a medical therapy for achondroplasia is available. In a field where interventions have until recently only addressed the complications of the condition, the advent of a medical therapy addressing the underlying pathophysiology will raise many questions on how current interventions may be impacted, and on the safety and efficacy of combined approaches. Collecting data on the combination of vosoritide and limb surgery will be important to assess the safety and efficacy of the dual treatment approach, however, while data are lacking, individual decision-making based on the physician’s experience integrating the medical knowledge and all available data alongside the values and preferences of the patient and family will be necessary. Comparison of data from three subgroups (vosoritide alone, surgical lengthening alone, vosoritide plus surgical lengthening) vs. a group with no interventions for height would be beneficial to enable comparison of indices such as lengthening (cm/month), consolidation time after completion of lengthening, and percentage of growth achieved.

While evidence-based data are not available, collating and sharing expert opinion is a vital way of providing support and guidance to the clinical community.

## Electronic supplementary material

Below is the link to the electronic supplementary material.

Supplementary Material 1

Supplementary Material 2

## Data Availability

For full transparency all data supporting the findings of this Delphi process are available within the paper.

## Abbreviations

AGV: annualised growth velocity
CNP: C-type natriuretic peptide
ENT: ear, nose, and throat
FMS: foramen magnum stenosis
LL: limb lengthening
LoA: level of agreement
MDT: multidisciplinary team
