# Supplementary material for: International expert opinion on the considerations for combining vosoritide and limb surgery: a modified delphi study
Source: Orphanet J Rare Dis. 2024 Sep 17;19:347. doi: 10.1186/s13023-024-03236-4 (PMC11409630; doi:10.1186/s13023-024-03236-4)
Supplement: Supplementary file 1 — Supplementary Material 1 [file 13023_2024_3236_MOESM1_ESM.docx]

Julia Vodopiutz

Vienna Bone and Growth Center,
Währinger Gürtel 18–20,
1090 Vienna,
Austria
[julia.vodopiutz@meduniwien.ac.at](mailto:julia.vodopiutz@meduniwien.ac.at)

**International Expert Opinion on the Considerations for Combining Vosoritide and Limb Surgery: A Modified Delphi Study**

Dear Sir/Madam,

I submit for your consideration ‘*International Expert Opinion on the Considerations for Combining Vosoritide and Limb Surgery: A Modified Delphi study’.*

With the medical therapy, vosoritide, now available for the treatment of achondroplasia, there are many questions among the clinical community about how this new therapy may work alongside or in combination with limb surgery, a common option for the management of complications associated with the condition. An international expert panel of 17 clinicians and orthopaedic surgeons was convened, and a modified Delphi process undertaken to assess the considerations for combining limb surgery and vosoritide in four sections: The knowns and unknowns of vosoritide, limb surgery, and combination of the two, that should be taken into consideration in all treatment planning and decision-making; Considerations for patients who may accept either vosoritide or limb surgery, separately, or in any combination; Considerations for patients who have already started limb surgery where vosoritide is being considered; and Considerations for patients already prescribed vosoritide where limb surgery is being considered.

A modified Delphi process was carried out and 97 statements were rated on a ten-point scale where 1 was ‘Completely disagree’ and 10 ‘Completely agree’. This manuscript is the first to provide some guidance, based on expert opinion, on the use of vosoritide and limb surgery in combination, and will be valuable in answering the questions that the clinical community have on this topic.

I hope you will consider it for publication in *Orphanet Journal of Rare Diseases*.

The content of this manuscript has not been published or submitted for publication elsewhere.

I can confirm that all authors have contributed to and approved the final manuscript for publication.

Author email addresses and any conflicts of interest are outlined on the next page.

I look forward to your response.

Kind regards,

Dr Julia Vodopiutz

**Authors**

- Silvio Boero, Pediatric Orthopaedic and Traumatology Unit, Istituto Giannina Gaslini, Genoa, Italy

Email: [silvioboero@gaslini.org](mailto:silvioboero@gaslini.org)

Conflict of interest: has received payment or honoraria, support for attending meetings and/or travel, and has participated on a Data Safety Monitoring Board or Advisory Board from BioMarin.

- Julia Vodopiutz, Vienna Bone and Growth Center, Vienna, Austria, Währinger Gürtel 18–20, 1090 Vienna, Austria; and Department of Pediatrics and Adolescent Medicine, Division of Pediatric Pulmonology, Allergology and Endocrinology, Comprehensive Center for Pediatrics, Medical University of Vienna, 1090 Vienna, Austria

Email: [julia.vodopiutz@meduniwien.ac.at](mailto:julia.vodopiutz@meduniwien.ac.at)

Conflict of interest: has received payment or honoraria from BioMarin

- Mohamad Maghnie, Department of Pediatrics, IRCCS Istituto Giannina Gaslini, 16147, Genova, Italy, and Department of Neuroscience, Rehabilitation, Ophthalmology, Genetics, Maternal and Child Health, University of Genova, 16147, Genova, Italy

Email: [MohamadMaghnie@gaslini.org](mailto:MohamadMaghnie@gaslini.org)

Conflict of interest: has received grants or contracts from Pfizer, Novo Nordisk, and Merck Serono (paid to IRCCS Istituto Giannina Gaslini and DINOGM); Consulting fees and payment or honoraria from Merck Serono, Novo Nordisk, Pfizer, Sandoz, and BioMarin; and has participated on a Data Safety Monitoring Board or Advisory Board from Pfizer, Merck, Novo Nordisk and BioMarin

- Josep M. de Bergua, Unidad Cirugía Artroscópica (UCA), Hospital Vithas Vitoria, Vitoria-Gasteiz, Spain

Email: [jmdebergua@gmail.com](mailto:jmdebergua@gmail.com)

Conflict of interest: has received payment or honoraria from BioMarin

- Ignacio Ginebreda, Hospital Universitari Dexeus - Grupo Quirónsalud, Calle Sabino Arana, 5-19 - Planta 1 08028 Barcelona, Spain

Email: [Ignacio.ginebreda@icatme.com](mailto:Ignacio.ginebreda@icatme.com)

Conflict of interest: has received payment for expert testimony, support for attending meetings and/or travel, has participated on a Data Safety Monitoring Board or Advisory Board and has a leadership or fiduciary role, all paid to his institution

- Hiroshi Kitoh, Department of Orthopaedic Surgery, Aichi Children’s Health and Medical Center 7-426, Morioka-cho, Obu, Aichi 474-8710, Japan

Email: [hkitoh420@gmail.com](mailto:hkitoh420@gmail.com)

Conflict of interest: has no conflicts of interest to declare

- Micha Langendörfer, Orthopedic Department of Kinderklinik Sankt Augustin, Arnold-Janssen-Straße 29, 53757 St. Augustin, Germany

Email: [m.langendoerfer@asklepios.com](mailto:m.langendoerfer@asklepios.com)

Conflict of interest: has received grants or contracts and consultancy fees from Smith Nephew, Nuvasive, and Stryker

- Antonio Leiva-Gea, UGC Cirugía Ortopédica y Traumatología, Hospital Universitario Virgen de la Victoria, Instituto de Investigación Biomédica de Málaga (IBIMA)-Plataforma Bionand, Málaga, España

Email: [antonioleiva7@yahoo.es](mailto:antonioleiva7@yahoo.es)

Conflict of interest: has received payment or honoraria, and support for attending meetings and/or travel from MBA-Orthofix and BioMarin

- Jason Malone, Nemours Children’s Hospital – Florida, Orlando, Florida, United States

Email: [Jason.malone@nemours.org](mailto:Jason.malone@nemours.org)

Conflict of interest: has no conflict of interest to declare.

- Philip McClure, Rubin Institute for Advanced Orthopedics, Sinai Hospital of Baltimore 2401 W. Belvedere Avenue, Baltimore, Maryland 21215 USA

Email: [Pmcclure@lifebridgehealth.org](mailto:Pmcclure@lifebridgehealth.org)

Conflict of interest: has received consultancy fees from Orthofix, Novasure, Wishbone, Novadip, Synthes and Smith/Nephew.

- Gabriel T. Mindler, Vienna Bone and Growth Center, Vienna, Austria, Währinger Gürtel 18–20, 1090 Vienna, Austria; and Department of Pediatric Orthopaedics and Foot Surgery, Orthopaedic Hospital Speising, Speisinger Strasse 109, 1130 Vienna, Austria

Email: [Gabriel.Mindler@oss.at](mailto:Gabriel.Mindler@oss.at)

Conflict of interest: has received consulting fees from Kyowa Kirin and BioMarin; payment or honoraria from Kyowa Kirin and has participated on a Data Safety Monitoring Board or Advisory Board for BioMarin and Kyowa Kirin.

- Dmitry Popkov, National Ilizarov Medical Research Centre for Traumatology and Ortopaedics, 6, M.Ulyanova street, Kurgan, 640014, Russia

Email: [dpopkov@mail.ru](mailto:dpopkov@mail.ru)

Conflict of interest: has no conflicts of interest to declare

- Robert Rodl, Universitätsklinikum Münster, Universitätsklinikum Münster Albert-Schweitzer-Campus 1, Gebäude A1, Anfahrtsadresse: Albert-Schweitzer-Straße 33, 48149 Münster

Email: [roedlr@ukmuenster.de](mailto:roedlr@ukmuenster.de)

Conflict of interest: has received royalties from Merete, payment or honoraria from Nuvasive, Smith Nephew, Infectopharm, FOMF, VKO and Kyowa Kirin; patents planned, issued or pending with Merete and has participated on a Data Safety Monitoring Board or Advisory Board for Kyowa Kirin and BioMarin

- Pablo Rosselli, Fundación Cardio infantil Facultad de Medicina, Bogota, Colombia

Email: [dr.rosselli@gmail.com](mailto:dr.rosselli@gmail.com)

Conflict of interest: has received payment or honoraria, support for attending meetings and/or travel, and has participated on a Data Safety Monitoring Board or Advisory Board for BioMarin

- Fabio Verdoni, IRCCS ‘Galeazzi’ Orthopedic Institute, Vis Riccardo Galeazzi, 4 20161 Milano, Italy

Email: [verdonifabio@gmail.com](mailto:verdonifabio@gmail.com)

Conflict of interest: has received payment or honoraria from BioMarin, and has participated on a Data Safety Monitoring Board or Advisory Board for Biomedical Pharma

- Viktor Vilenskii, St Petersburg State University Hospital, St Petersburg, Russia

Email: [vavilensky@mail.ru](mailto:vavilensky@mail.ru)

Conflict of interest: has no conflicts of interest to declare

- Aaron J. Huser, Paley Advanced Limb Lengthening Institute, West Palm Beach, Florida, United States

Email: [ahuser@paleyinstitute.org](mailto:ahuser@paleyinstitute.org)

Conflict of interest: has received payments for speaker bureaus, and has participated on a Data Safety Monitoring Board or Advisory Board, from BioMarin
