# Supplementary material for: International expert opinion on the considerations for combining vosoritide and limb surgery: a modified delphi study
Source: Orphanet J Rare Dis. 2024 Sep 17;19:347. doi: 10.1186/s13023-024-03236-4 (PMC11409630; doi:10.1186/s13023-024-03236-4)
Supplement: Supplementary file 2 — Supplementary Material 2 [file 13023_2024_3236_MOESM2_ESM.docx]

**International Expert Opinion on the Considerations for Combining Vosoritide and Limb**

**Surgery: A Modified Delphi Study**

**Response to reviewers**

Dear Sir/Madam,

Thank you for your kind review of our manuscript and your suggestions for improvement and clarification. Please see below a response to the reviewer’s comments. Appropriate changes have been made in the accompanying revised manuscript.

**Reviewer 1**

**Comments:**

Dear Author,
Your manuscript is well written and results are well supported by data and conclusion however the manuscript needs to be edited by native English speaker for English Language and grammar

**Response**

Thank you, the manuscript has previously been reviewed by a native English speaker, however another native English speaker has reviewed and made some minor changes.

**Reviewer 2**

**Comments:**

Nicely written

**Response**

Thank you for your comments
